# Supplementary material for: Deep phenotyping of skin tissue remodeling in patients with systemic sclerosis treated with CD19-CAR T cells
Source: Nat Commun. 2026 May 23;17:4640. doi: 10.1038/s41467-026-72817-7 (PMC13201536; doi:10.1038/s41467-026-72817-7)
Supplement: Supplementary file 1 — Supplementary Information [file 41467_2026_72817_MOESM1_ESM.pdf]

Deep phenotyping of skin tissue remodeling in patients with systemic sclerosis treated with CD19-CAR T cells

- Supplementary figures and tables -

Aleix Rius Rigau*, Meilin Xu*, Ziyuan Liu*, Sara Chenguiti Fakhouri* 1,2, Janina Auth, Panagiotis Garantziotis, Andrea Zoli, Manoj Kumar, Maria Gabriella Raimondo1, Carlo Tur, Tim Filla, Paula Gehringer, Markus Eckstein, Fabian Müller, Armin Atzinger, Moritz Ronicke, Arif Ekici, Rafael Schmid, Andreas Wirsching, Melanie Hagen, Sebastian Böltz, Tobias Krickau, Raymund E. Horch, Carola Berking, Ricardo Grieshaber-Bouyer, Andreas Ramming, Pooja Gupta, Aline Bozec, Andreas Mackensen, Jörg HW Distler, Georg Schett, Yi-Nan Li+ and Christina Bergmann+

* contributed equally, + supervised equally

Corresponding author: Christina Bergmann, MD; Department of Internal Medicine 3 - Rheumatology and Immunology, Friedrich-Alexander-Universität Erlangen-Nürnberg and Uniklinikum Erlangen, Erlangen, Germany, Phone: +49 9131 43114, FAX: +49 9131 36448, Email: christina.bergmann@uk-erlangen.de

List of supplementary figures:

**Supplementary Figure 1**:	Analytical overview of deep tissue phenotyping in skin
**Supplementary Figure 2**:	Changes in peripheral blood upon CD19-CAR T cell therapy
**Supplementary Figure 3**:	Depletion of B cells in skin tissue following CD19-CAR T cell therapy
**Supplementary Figure 4**:	CAR T transcriptomic response among skin cells measured by cISH and lung function parameters of patients
**Supplementary Figure 5**:	Pulmonary FAPI-uptake before and after CD19-CAR T-cell therapy
**Supplementary Figure 6**:	Skin gene expression patterns upon CD19-CAR T cell therapy
**Supplementary Figure 7**:	Gene expression pattern in papillary and reticular dermis after CD19-CAR T cell therapy
**Supplementary Figure 8**:	Spatial localization of fibroblast populations identified by cISH
**Supplementary Figure 9**:	Correspondence of cISH-identified fibroblasts to skin fibroblast atlas
**Supplementary Figure 10:**	Functional phenotypes of the fibroblast populations detected by cISH
**Supplementary Figure 11**:	Temporal analysis of cell composition after CD19-CAR T therapy

Supplementary Figure 12: Fibroblast composition in papillary and reticular dermis after CD19-CAR T therapy
Supplementary Figure 13: Identification of cell types by IMC
Supplementary Figure 14: Main skin cell types dynamics across different time points
Supplementary Figure 15: Fibroblast dynamics across the time points and their spatial dermal distribution
Supplementary Figure 16: Fibroblast composition after CD19-CAR T-cell therapy is similar to non-diseased
Supplementary Figure 17: Correlation of fibroblast clusters percentage increase with clinical outcome
Supplementary Figure 18: IMC fibroblast marker expression in cISH dataset
Supplementary Figure 19: Endothelial cell populations identified by cISH
Supplementary Figure 20: Epithelial populations identified by cISH

Supplementary Table 1: Baseline characteristics of patients with diffuse systemic sclerosis before CD19-targeting CAR T cell therapy, standard-of-care (SOC) treatments or with natural disease course (NDC)

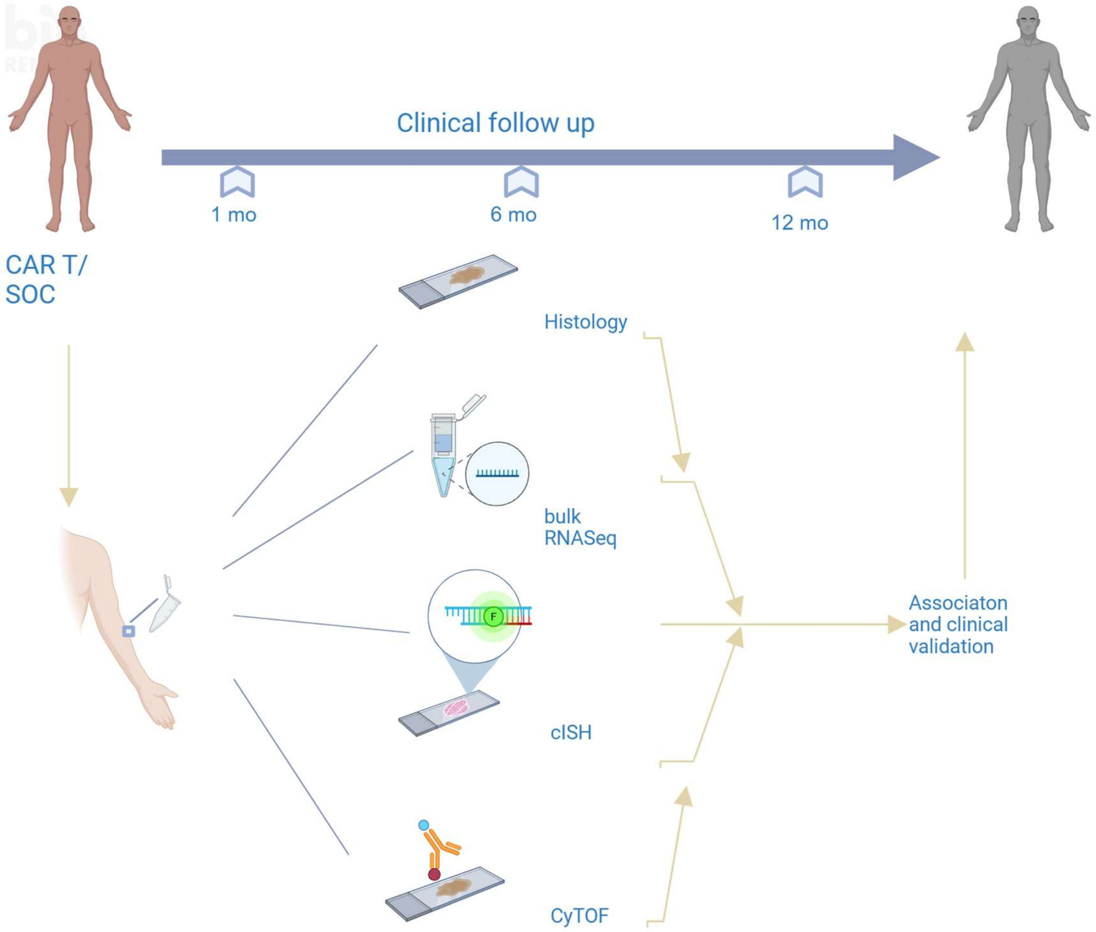

Supplementary Figure 1: Analytical overview of deep tissue phenotyping in skin.

The skin biopsies were collected from patients who received CD19-CAR T cell therapy or standard-of-care (SOC) treatment at baseline and specific follow-up time points. The tissue phenotyping was performed in a multi-modal manner with four approaches: (1) histopathological analysis; (2) transcriptome profiling using RNA-Seq; (3) cISH-based spatial transcriptomics; (4) IMC-based spatial proteomics. (5) The multi-omic results were further analyzed for assiociation with clinical parameters. This figure was created in Biorender. . Bergmann, C. (2026) https://BioRender.com/siutwqe.

Baseline characteristics	CD19-CAR T	SOC	p-value
age (mean(SD))	41.5 (14.8)	46.6 (14.1)	0.46
Sex			
male (n (%))	7 (63.6)	5 (62.5)	0.94
female (n (%))	4 (36.4)	3 (37.5)	
Disease Duration (mo, mean (SD))	45.6 (37.4)	47.3 (59.0)	0.5
auto-antibody status			0.25
anti-Scl70 (n (%))	9 (81.8)	5 (62.5)	
anti-RNAP III (n (%))	1 (9.1)	0 (0)	
other (n (%))	1 (9.1)	3 (27.2)	
mRSS (mean (SD))	23.91 (6.2)	15.6 (4.6)	0.0072
Lung Involvement (n (%))	11 (100)	8 (100)	>0.99
Heart Involvement (n (%))	5 (45.5)	2 (25)	0.633
EUSTAR AI (mean (SD))	5.5 (2.0)	4.2 (1.6)	0.13
number of previous treatments (mean (SD))	3.1 (1.5)	0.9 (0.4)	0.0018

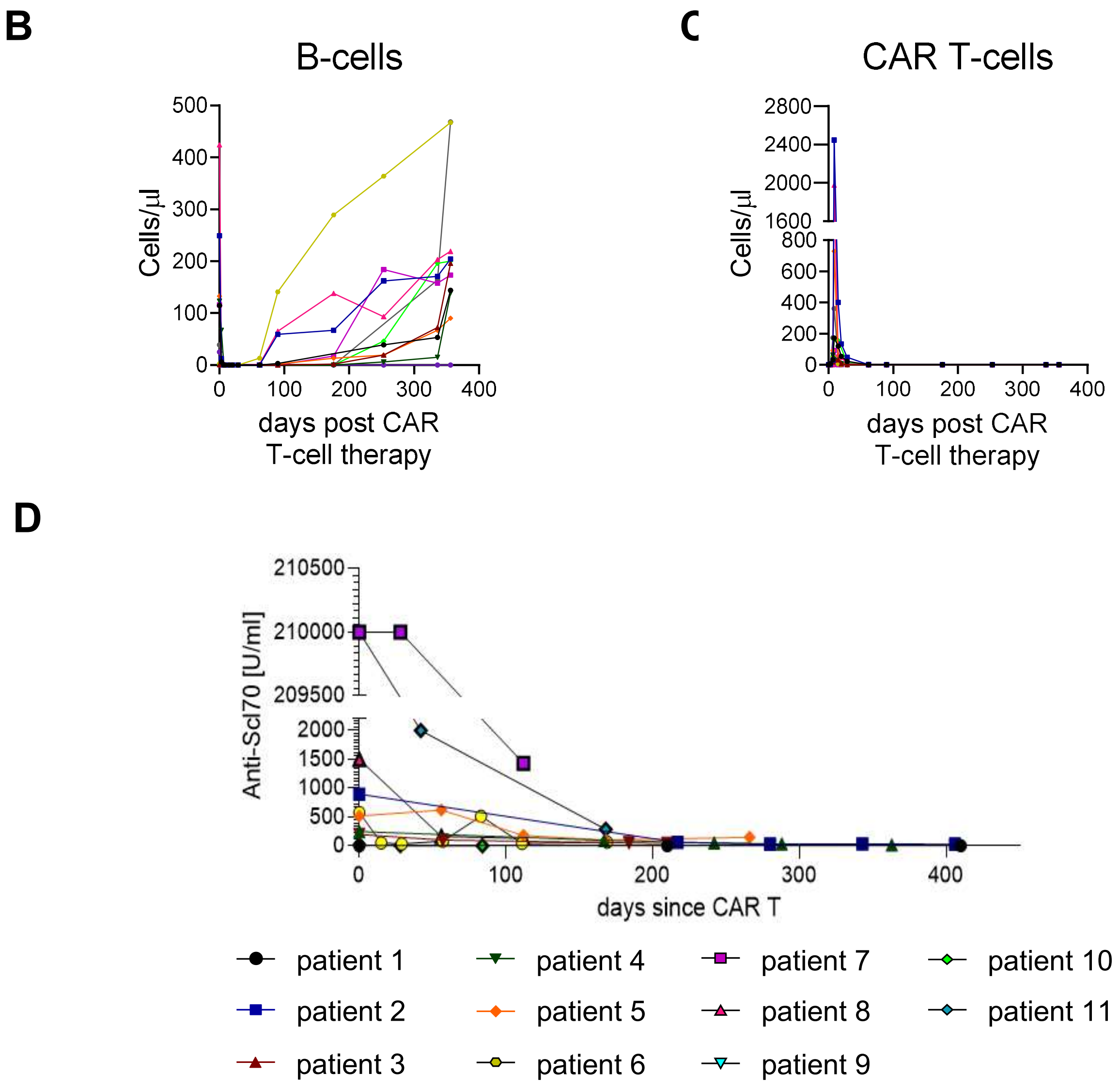

Supplementary Figure 2: Changes in peripheral blood upon CD19-CAR T cell therapy. A Summary of baseline characteristics of all participants. P-values < 0.005 were considered significant after Bonferroni-correction. **B** Individual course of the B cells numbers in peripheral blood after CD19-CAR T-cell therapy. **C** Individual course of the numbers of the CAR T-cells in peripheral blood after CD19-CAR T-cell therapy (A-C: patients 1 – 11 of the CAR T-cell therapy group). **D** Individual course of the Anti-Scl70 autoantibodies in peripheral blood, determined by ELISA, after CD19-CAR T-cell therapy.

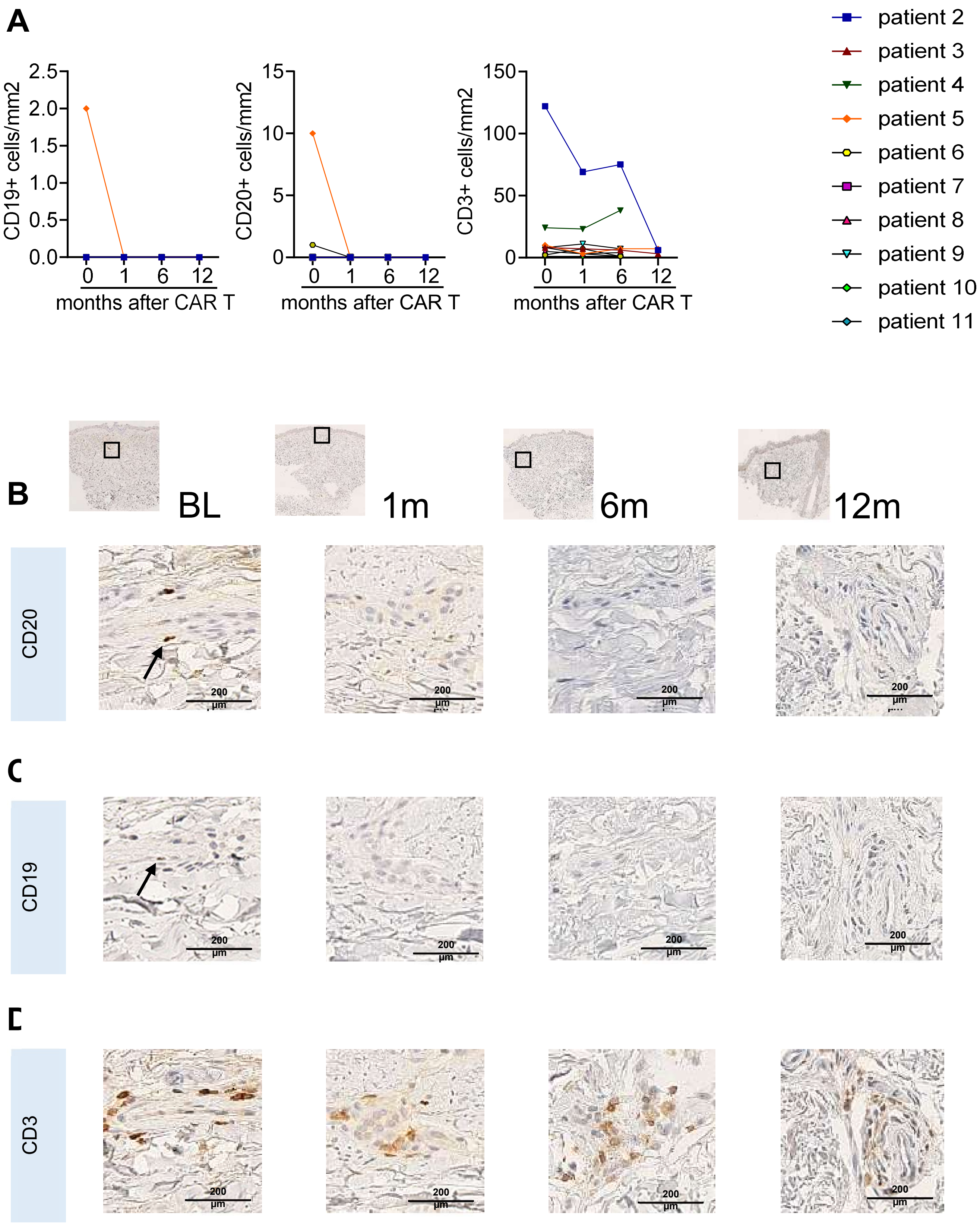

Supplementary Figure 3: **Depletion of B cells in skin tissue following CD19-CAR T cell therapy**. **A** Individual course of the numbers of CD19+, CD20+ B-cells and CD3+ T-cells before and after CD19-CAR T-cell treatment as analyzed by immunohistochemistry. **B C D** Representative immunochemistry staining images of the CD20 (B), CD19 (C) and CD3 (D) for the four timepoints. BL = Baseline; 1m = 1 month; 6m = 6 months; 12m = 12 months. All representative images are from patient 5 of the CD19-CAR T group.

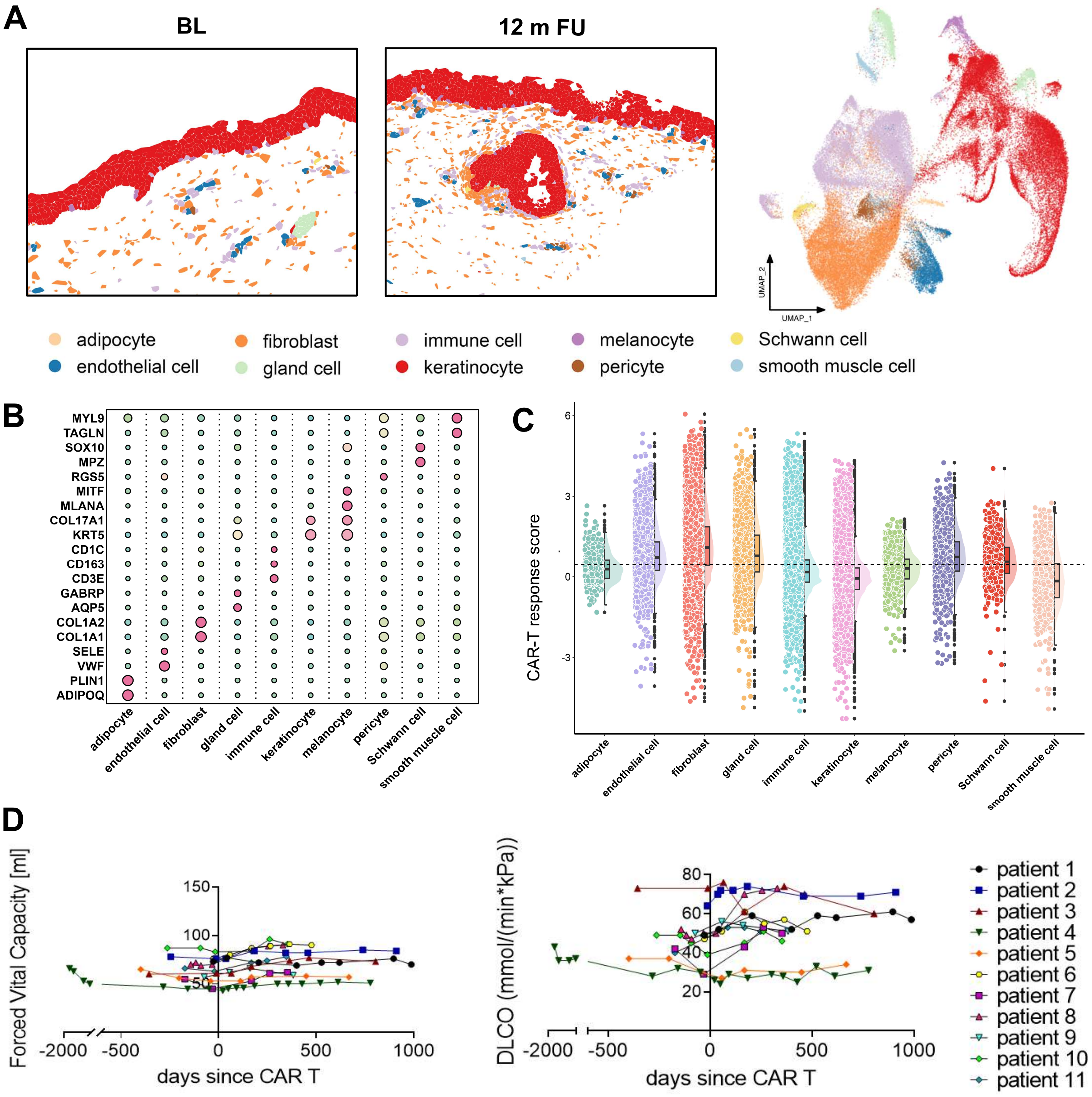

Supplementary Figure 4: CAR T transcriptomic response among skin cells measured by cISH and lung function parameters of patients. **A** Spatial images showing the localization of major cell types detected by cISH at baseline (BL; patient 5) and 12-month follow-up (12m FU; patient 1) skin tissue samples, respectively. UMAP plot depicts clustering of major cell types detected by cISH, including baseline and follow-up samples (n = 23). **B** Dot plot showing the expression of known marker genes in major cell types in skin. Dot size indicates the proportion of cells expressing each gene, dot color reflects the average expression level. **C** Raincloud plot showing the CAR T-cell response scores, derived from differentially expressed genes obtain by bulk RNA-Seq (1m FU vs BL), across different major cell types. The dashed line indicating the mean score, and each dot represents individual cell. **D** Development of lung function parameters before and after CD19-targeting CAR T-cell therapy: Forced vital capacity before and after CAR T-cell therapy; Diffusing capacity for carbon monoxide before and after CAR T-cell therapy.

A

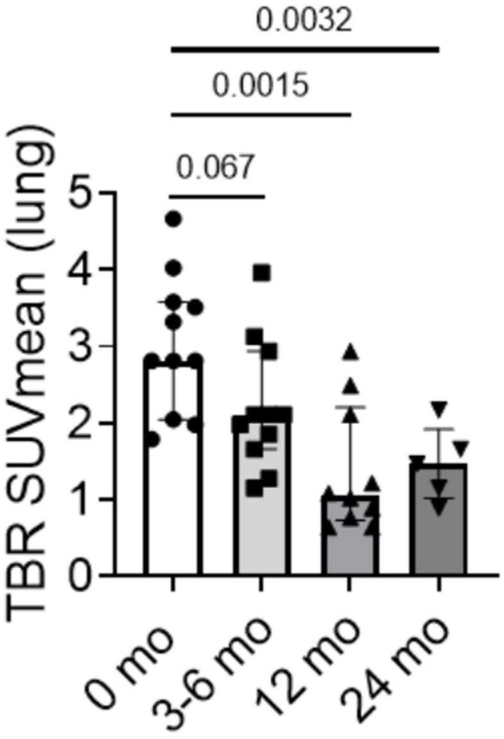

B

Time (mo)	BL	3-6 mo FU	12 mo FU	24 mo FU
Number of values	11	11	10	5
Minimum	1,790	1,151	0,6394	0,8951
25% Percentile	2,046	1,662	0,7353	1,023
Median	2,813	2,110	1,055	1,471
75% Percentile	3,581	2,941	2,206	1,918
Maximum	4,668	3,964	2,941	2,174
Range	2,877	2,813	2,302	1,279
Mean	3,034	2,209	1,381	1,471
Std. Deviation	0,8971	0,8343	0,8272	0,4911
Std. Error of Mean	0,2705	0,2516	0,2616	0,2196

Supplementary Figure 5: Pulmonary FAPI-uptake before and after CD19-CAR T-cell therapy. **A** Visualization of FAPI uptake (TBR SUVmean) by time point. Quantification across the cohort is visualized as bar graphs, median with interquartile range is shown, respectively. Paired comparisons were performed using Mann-Whitney-U Test. P-values < 0.016 were considered significant after Bonferroni correction. **B** Descriptive statistics.

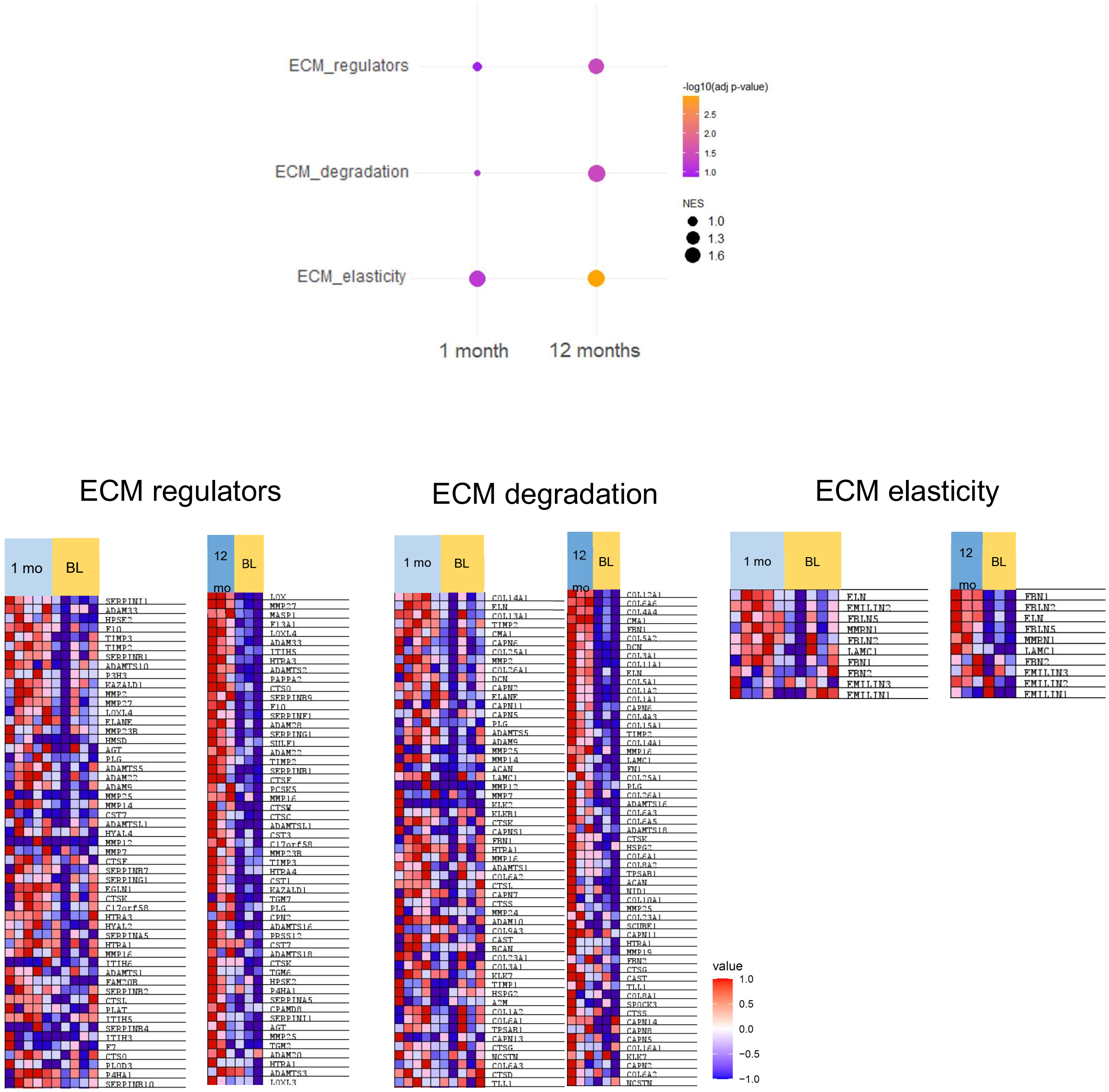

Supplementary Figure 6: Skin gene expression patterns upon CD19-CAR T cell therapy. Gene Set Enrichment Analysis (GSEA) enrichment dot plot and heatmaps of the leading-edge gene sets for extracellular matrix regulators (ECM) (Naba et al. 2012, Mol. Cell Proteomics), ECM degradation (R-HSA-1474228) and ECM elasticity-conferring constituents (GO:0030023) at 1 month (patients 1 to 5) and 12 month follow-up (patients 1 to 3). BL: Baseline; NES: Normalized enrichment score

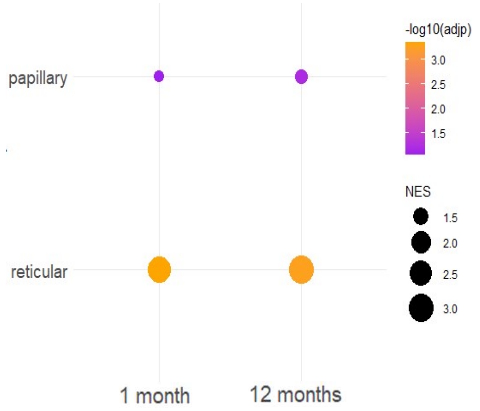

Papillary marker genes				
Follow-up	Set size	NES	pvalue	FDR qvalue
1 month	76	2.31755	<0.0001	<0.0001
12 months	76	1.4049298	<0.0001	<0.0001

Reticular marker genes				
Follow-up	Set size	NES	pvalue	FDR qvalue
1 month	141	3.1959069	<0.0001	<0.0001
12 months	141	3.0522754	0.034	0.035

Supplementary Figure 7: Gene expression pattern in papillary and reticular dermis after CD19-CAR T cell therapy. Gene Set Enrichment Analysis (GSEA) enrichment dot plot and enrichment score table of representative gene sets for papillary and reticular dermis marker genes (Solé-Boldo et al. 2020, Communications Biology) at 1 month and 12 month follow-up. BL: Baseline; NES: Normalized enrichment score.

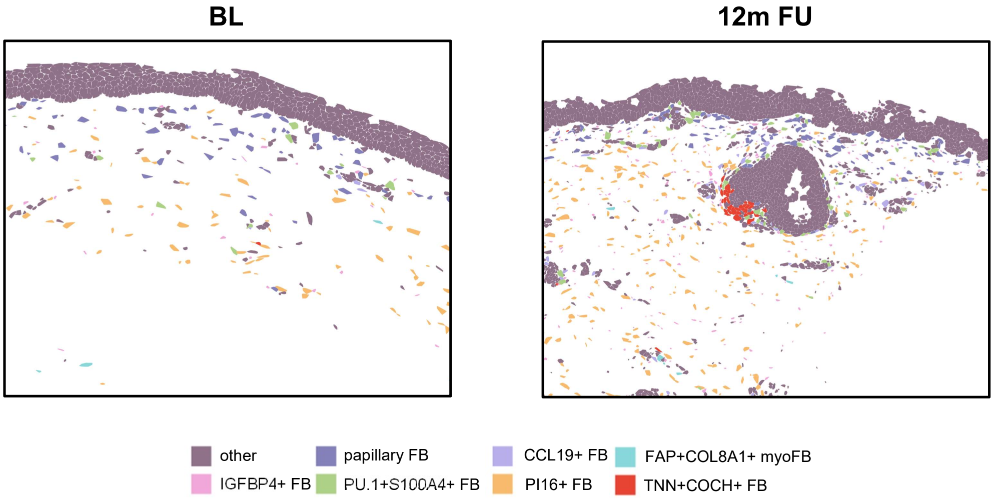

Supplementary Figure 8: Spatial localization of fibroblast populations identified by cISH. Representative spatial images showing the localization of fibroblast populations at baseline (BL; patient 6) and 12-month follow-up (12m FU; patient 1) in skin tissue. Colors represent the identity of fibroblast subpopulation. Cells other than fibroblasts are labelled as “other”.

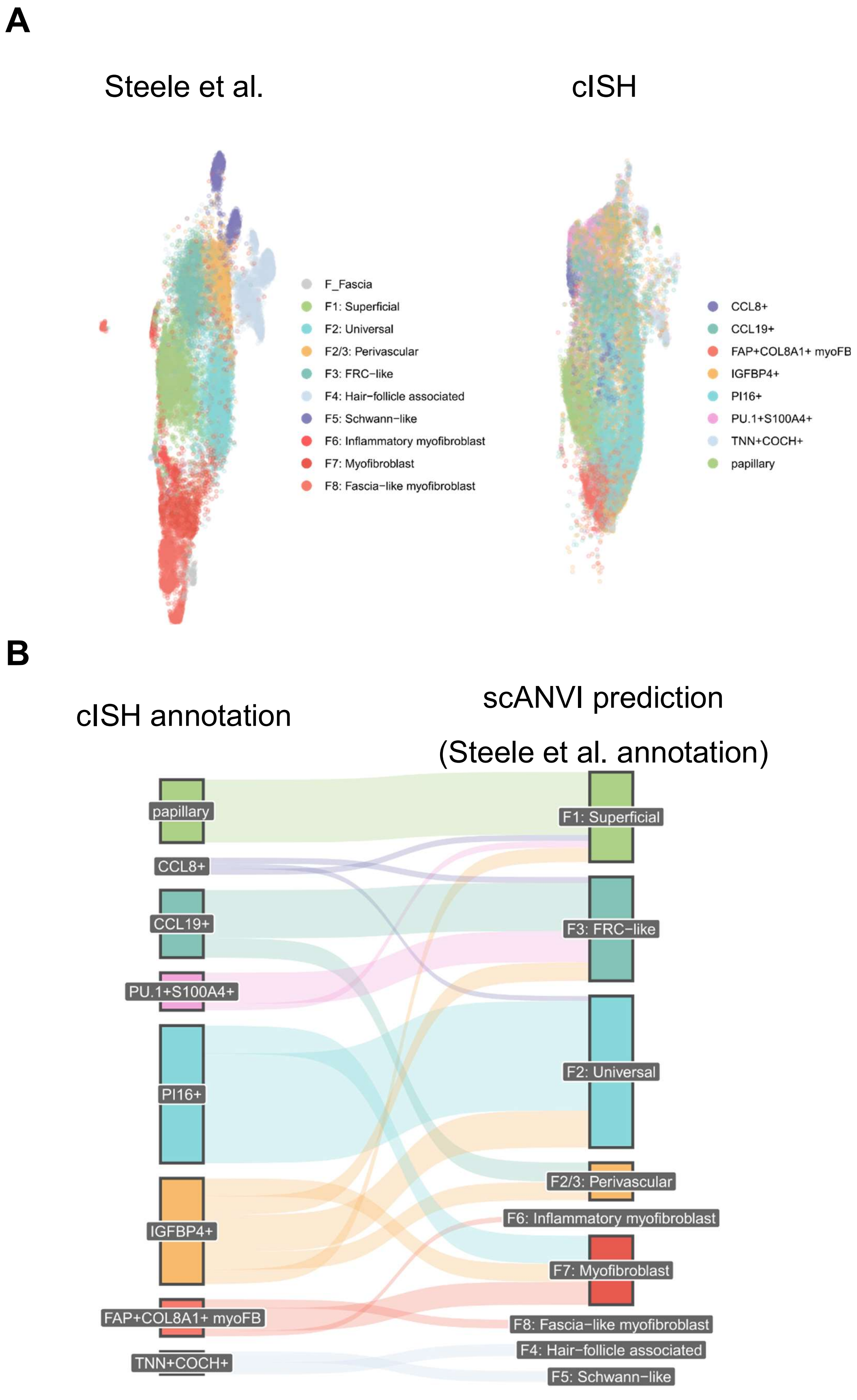

Supplementary Figure 9: Correspondence of cISH-identified fibroblasts to skin fibroblast atlas. **A** UMAP showing scANVI-based integration of fibroblast populations identified by cISH with the populations reported in the skin fibroblast atlas [42]. **B** Sankey plot showing the correspondence between cISH-identified fibroblasts and the populations defined in Steele et al., as predicted by scANVI.

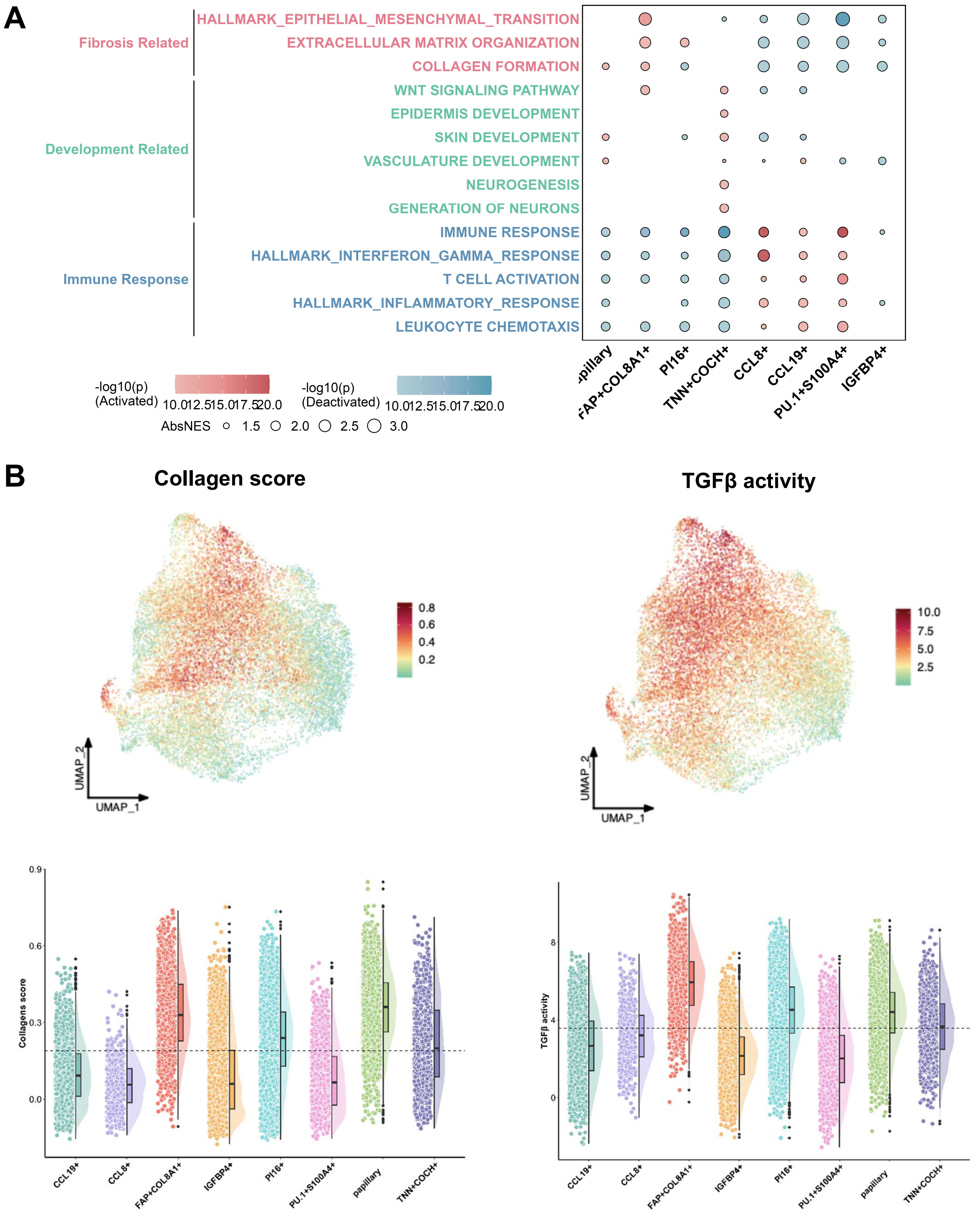

Supplementary Figure 10: Functional phenotypes of the fibroblast populations detected by cISH. A Dot plot showing enriched pathways detected by FGSEA across different fibroblast subtypes. Pathways are grouped into three functional categories—fibrosis-related, development-related, and immune response—and are colored accordingly. Dot size represents the absolute normalized enrichment score (AbsNES), while dot color reflects the log-transformed p-value. Red dots indicate activated pathways, and blue dots indicate deactivated pathways. **B** UMAP plots showing collagen score and PROGENy-inferred TGFβ activity across different fibroblast subtypes, based on the same UMAP embedding of cell types shown in Fig. 2D. Raincloud plots display collagen score and TGFβ activity for different fibroblast subtypes, with the dashed line representing the mean score/activity. Colors correspond to different fibroblast subtypes.

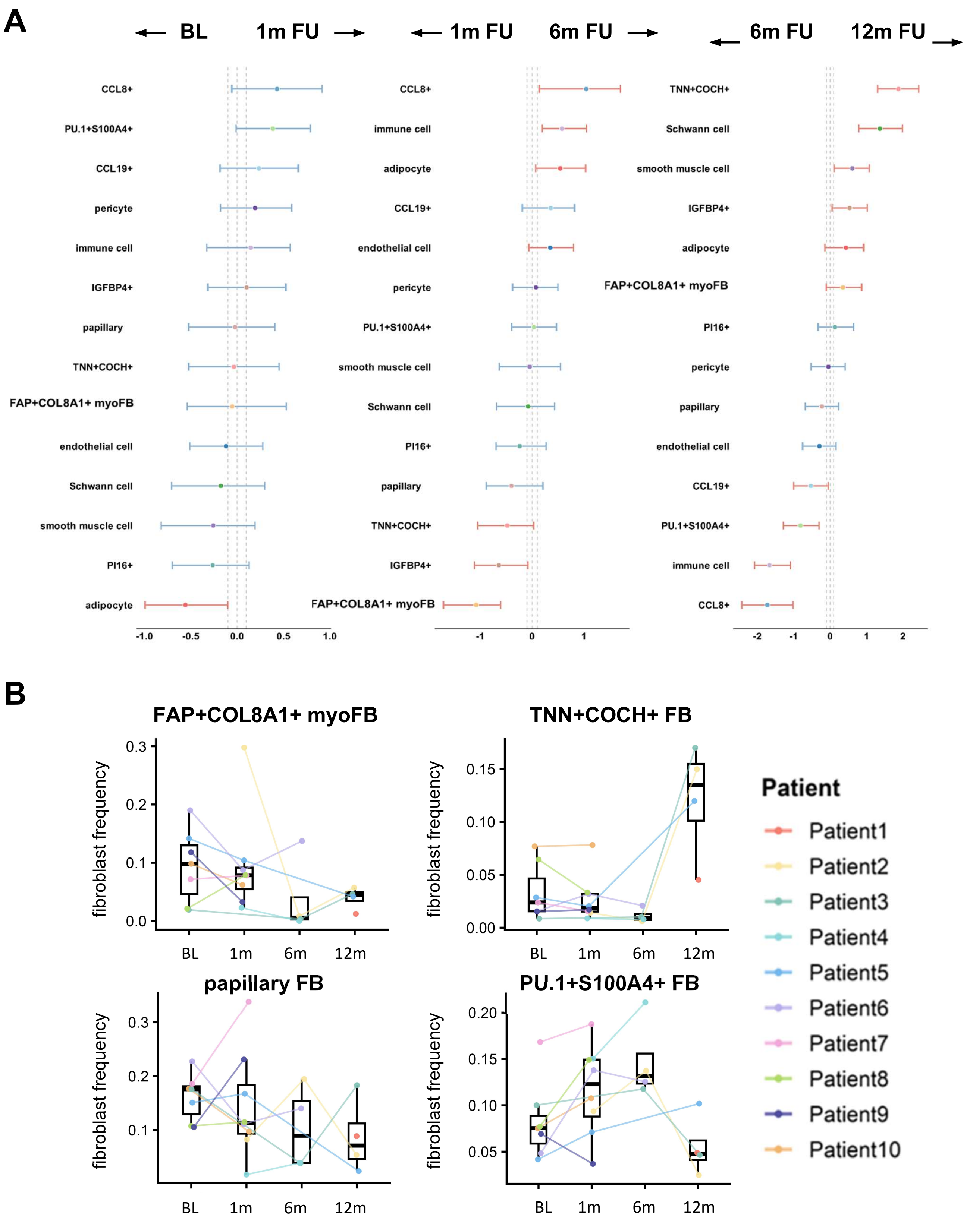

Supplementary Figure 11: **Temporal analysis of cell composition after CD19-CAR T therapy**. **A** Forest plots showing the differential composition of non-epithelial cells across time points: 1m FU vs. baseline, 6m FU vs. 1m FU, and 12m FU vs. 6m FU. Points represent mean posterior estimates (colored by cell type), with error bars indicating 95% credible intervals. Red bars indicate statistically significant changes (FDR < 0.05), and the dashed line represents the default threshold for the minimum effect size of ±0.1. **B** Boxplots showing the distribution of fibroblast frequencies at each timepoint. The x-axis represents the timepoints, and the y-axis represents the fibroblast frequency for the specified subtype. Each dot represents an individual sample, with colors distinguishing patient identity; within-patient measurements are connected to depict longitudinal trajectories.

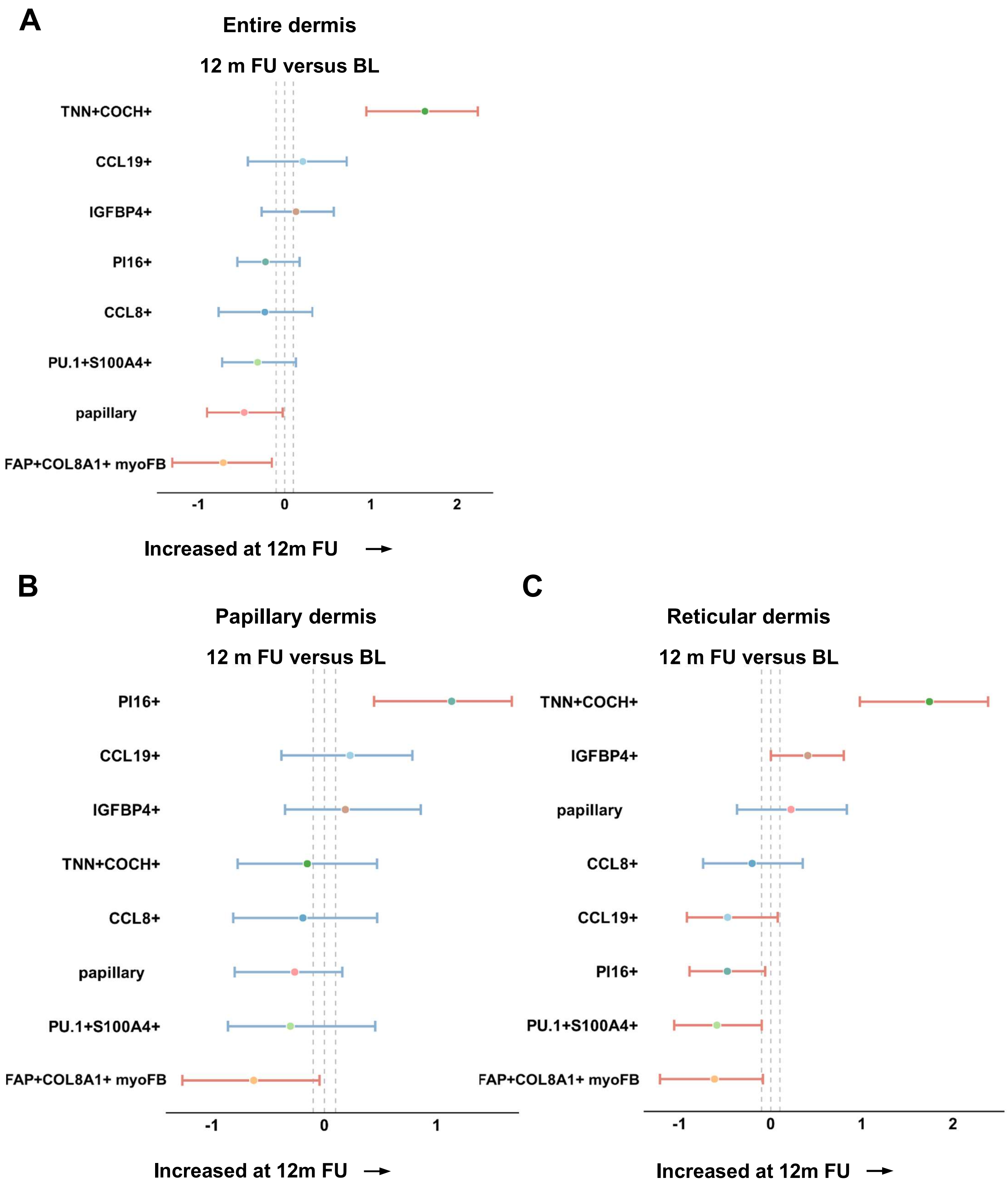

Supplementary Figure 12: **Fibroblast composition in papillary and reticular dermis after CD19-CAR T therapy.** Forest plots showing the differential composition of fibroblast subpopulations between baseline and 12m FU across different dermal compartments: entire dermis (A), papillary dermis (B), and reticular dermis (C). Points represent the mean posterior estimates (colored by fibroblast subpopulations), with error bars indicating 95% credible intervals. Red bars indicate significant changes (FDR < 0.05), including increased PI16+ and decreased FAP+COL8A1+ fibroblasts after CAR T therapy in papillary and reticular dermis, respectively. The dashed line represents the default threshold for the minimum effect size of ±0.1.

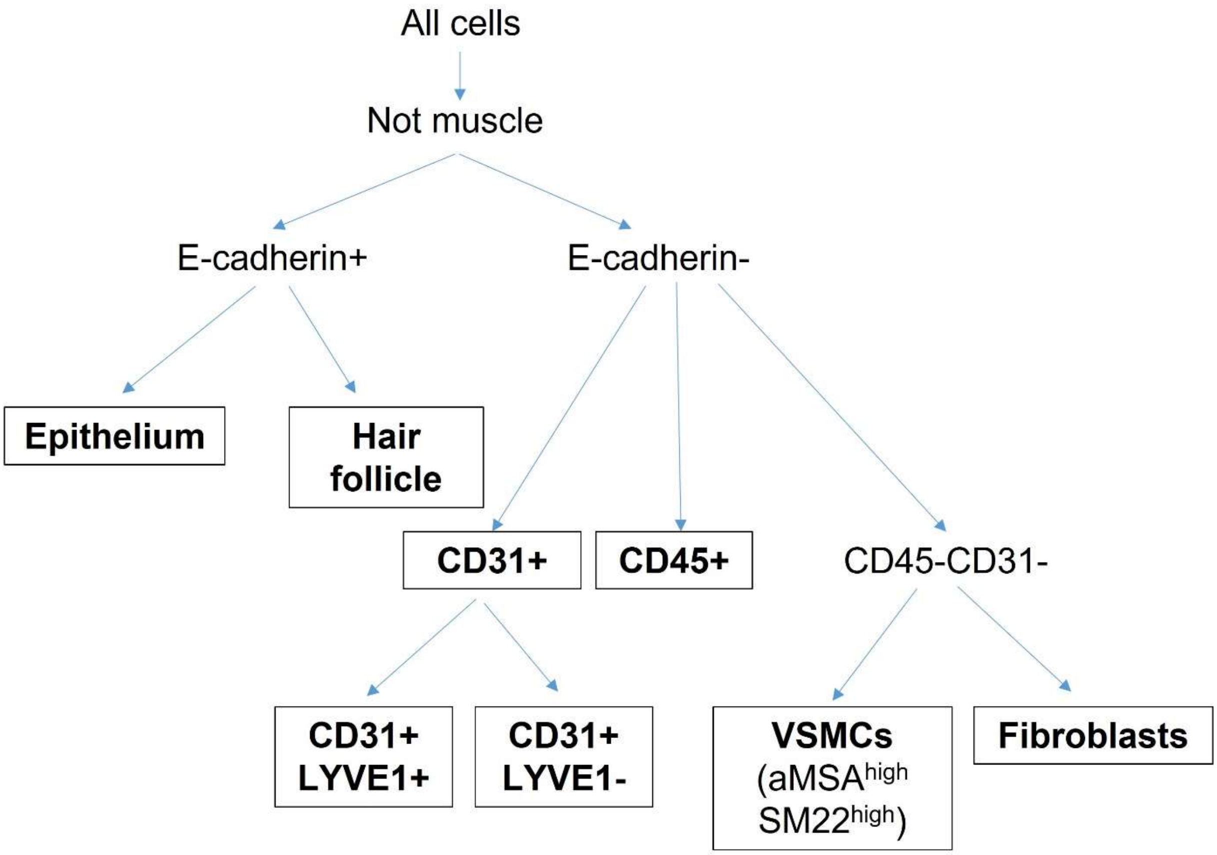

Supplementary Figure 13: Identification of cell types by IMC. Gating strategy for board cell type annotation for IMC data.

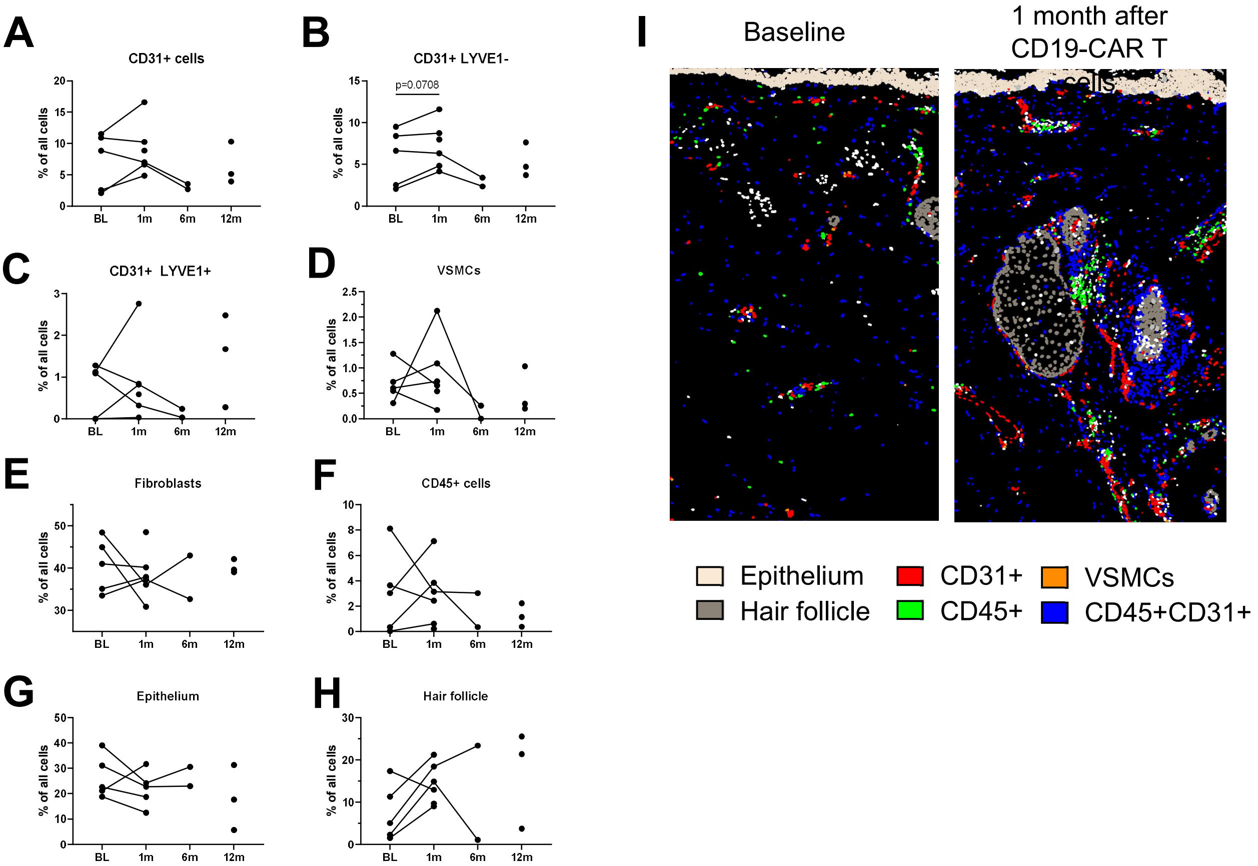

Supplementary Figure 14: Main skin cell types dynamics across different time points. A-H Changes of the main skin cell types across time points and showed as percentage of all cells. Each dot represents one sample (Baseline, n = 5; 1 months, n = 6; 6 months, n = 2; 12 months, n = 3). Statistical significance was determined by Paired t-test and P values are indicated above the comparisons if significance or trend is present. **I** Spatial distribution of the main skin cell types on the cell segmentation masks of two representative images at baseline (patient 5) and 1 month after CD19-CAR T-cell therapy (patient 8). BL = baseline; 1m = 1 month after infusion; 6m = 6 month after infusion; 12m = 12 month after infusion. VSMCs, Vascular smooth muscle cells.

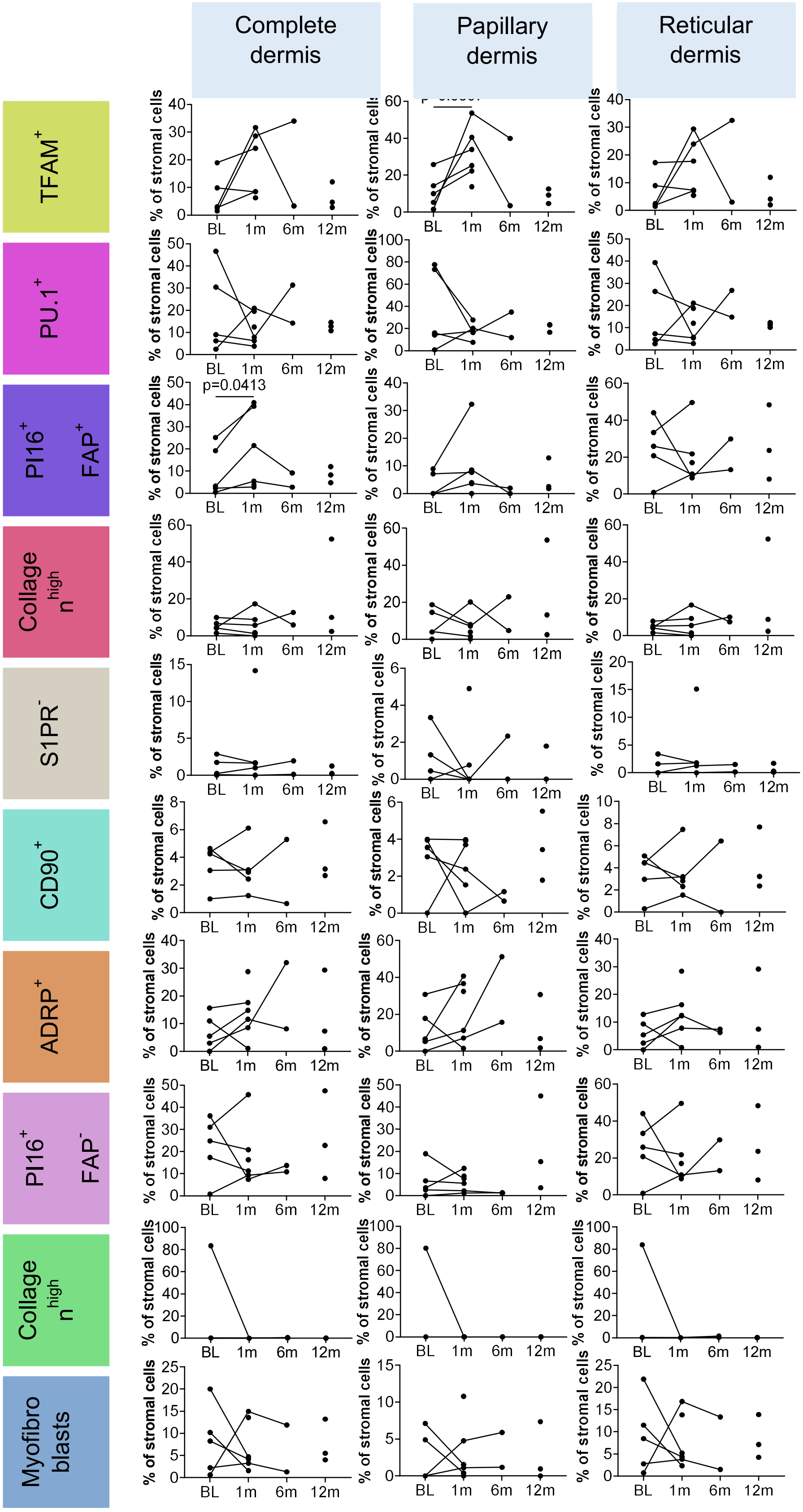

Supplemenary Figure 15: Fibroblasts dynamics across the time points and their spatial dermal distribution. Quantification of the 10 skin fibroblast clusters defined by IMC across the different time points in all dermis, papillary dermis or reticular dermis showed as percentage of total fibroblasts. Each dot represents one sample (Baseline, n = 5; 1 months, n = 6; 6 months, n = 2; 12 months, n = 3). Statistical significance was determined by Paired t-test and P values are indicated above the comparisons if significance or trend is present. BL = baseline; 1m = 1 month after infusion; 6m = 6 month after infusion; 12m = 12 month after infusion. Myofib.=myofibroblasts.

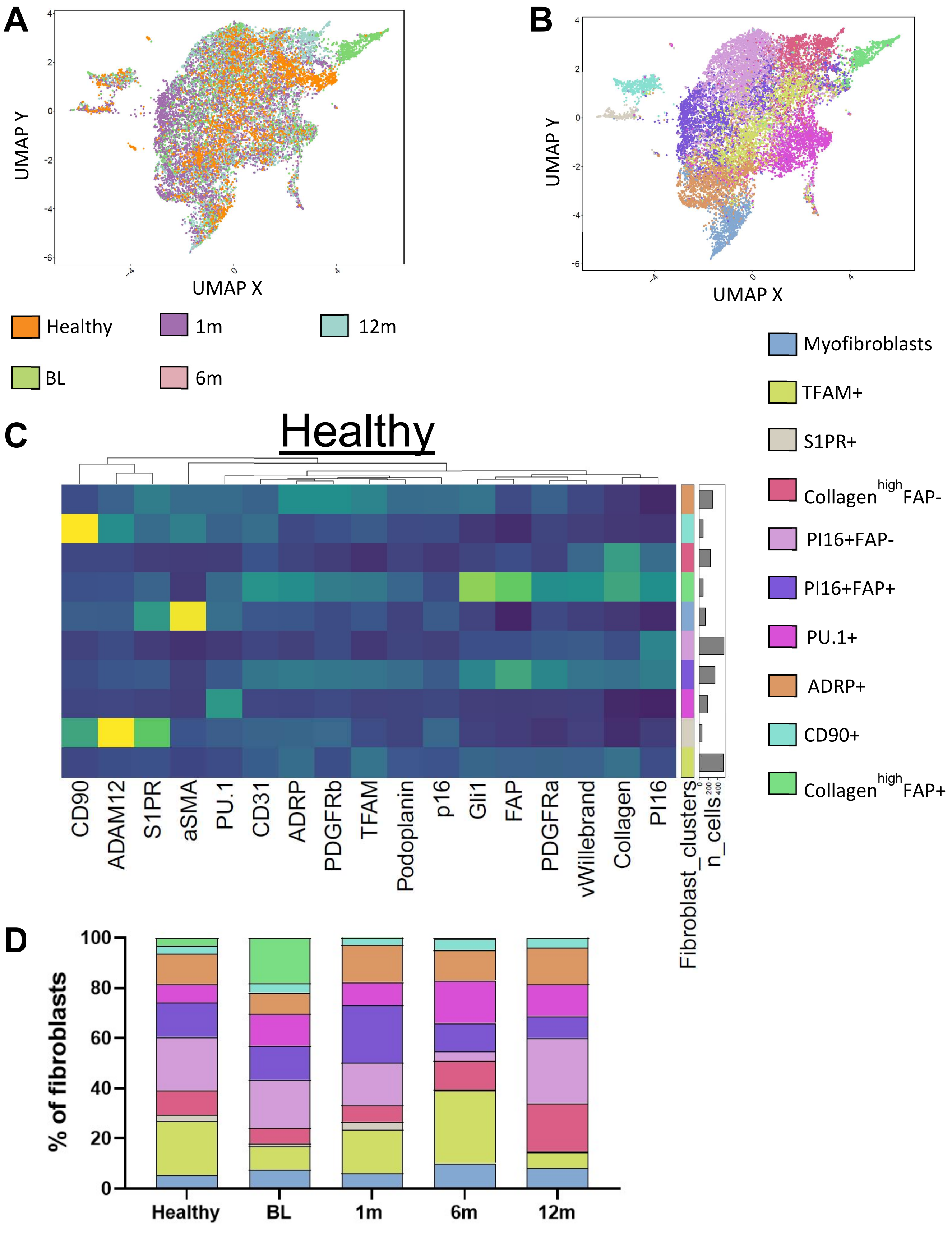

Supplementary Figure 16: Fibroblast composition after CD19-CAR T-cell therapy is similar to non-diseased. A UMAP plot showing fibroblasts of all patients (baseline, n = 5; 1 months, n = 6; 6 months, n = 2; 12 months, n = 3) and healthy controls (n = 7; 2 437 cells) based on the protein expression of all 22 shared markers colored by timepoint. **B** Same UMAP plot as in A but colored by fibroblast clusters. **C** Heatmap representing the protein expression of the fibroblast clusters of healthy data shown as z-score. The legend shows the number of cells, as indicated. **D Bar plot** showing the mean percentage of all fibroblasts for each fibroblast cluster in each timepoint. BL = baseline; 1m = 1 month after infusion; 6m = 6 month after infusion; 12m = 12 month after infusion

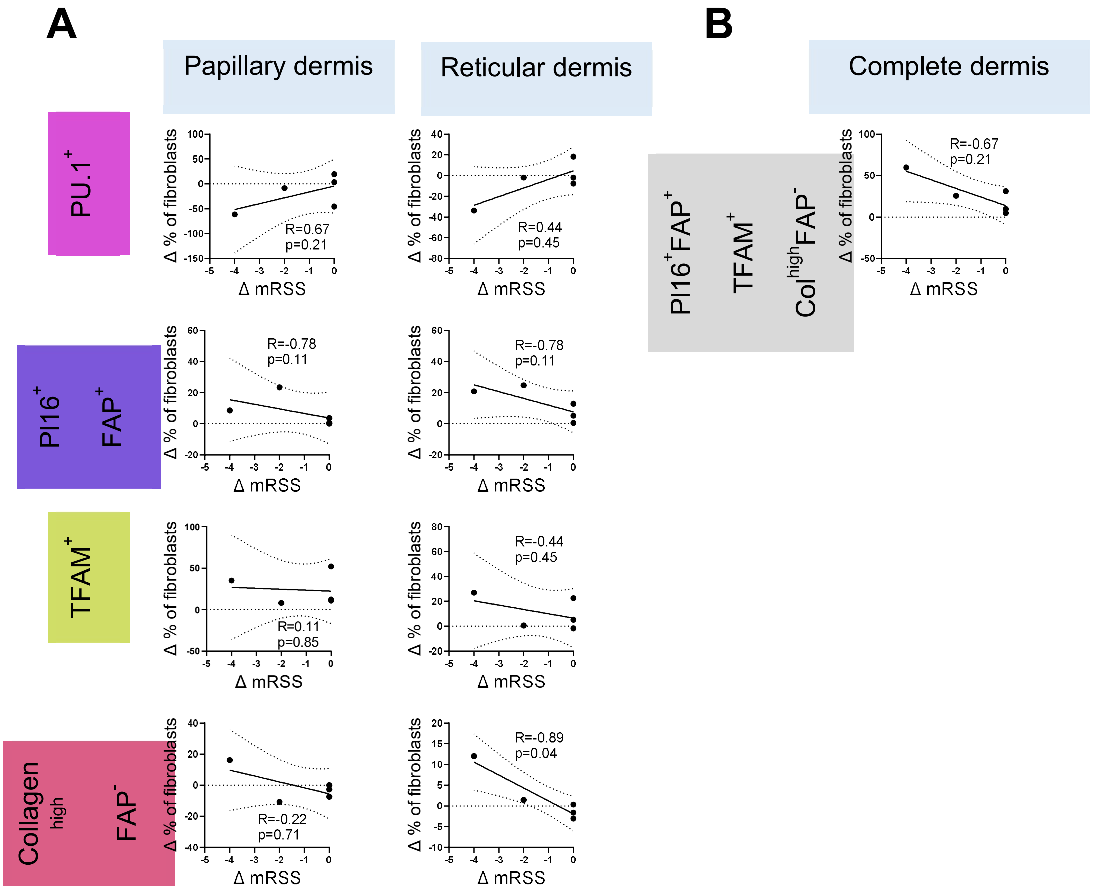

Supplementary Figure 17: Correlation of fibroblast clusters percentage increase with clinical outcome. A Spearman's correlation on the clinical outcome mRRS with the percentage of the selected fibroblast clusters on the papillary dermis and reticular dermis. **B** Spearman's correlation on the clinical outcome mRRS with the percentage of the merged 3 fibroblast clusters (PI16+FAP+, TFAM+ and Collagen+FAP-) on the whole dermis. All values represent the difference between 1 month and baseline (n=5). P- and R-values are indicated. BL = baseline

A

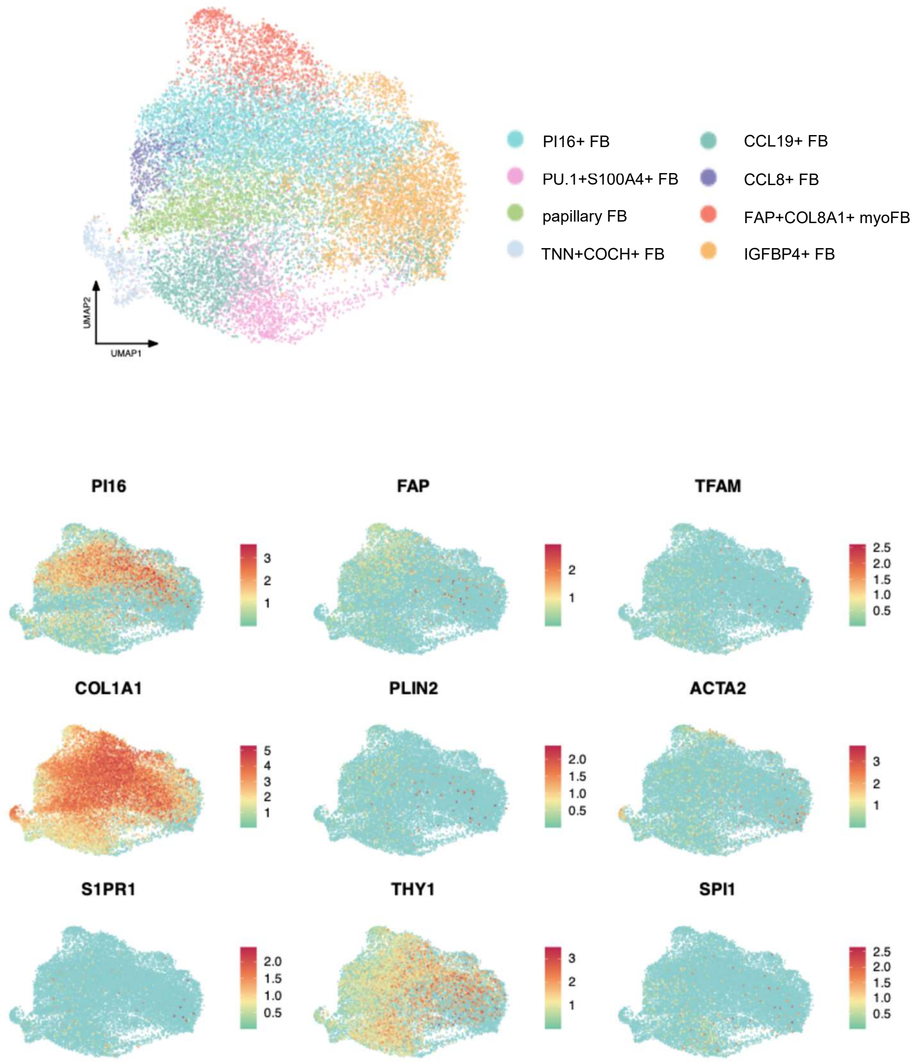

B

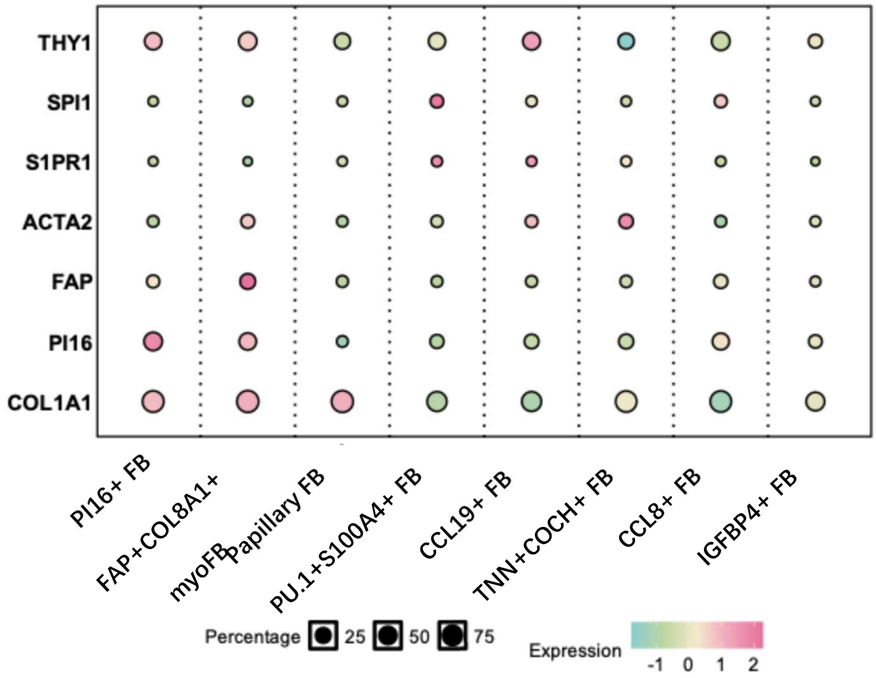

Supplementary Figure 18: IMC fibroblast marker expression in cISH dataset. A UMAP plots showing the expression of genes encoding IMC fibroblast markers for fibroblast subpopulations, based on the same UMAP embedding of fibroblast subtypes as shown. **B** Dot plot showing the expression of genes encoding IMC fibroblast markers for fibroblast subpopulations. Dot size indicates the proportion of cells expressing each gene, dot color reflects the average expression level.

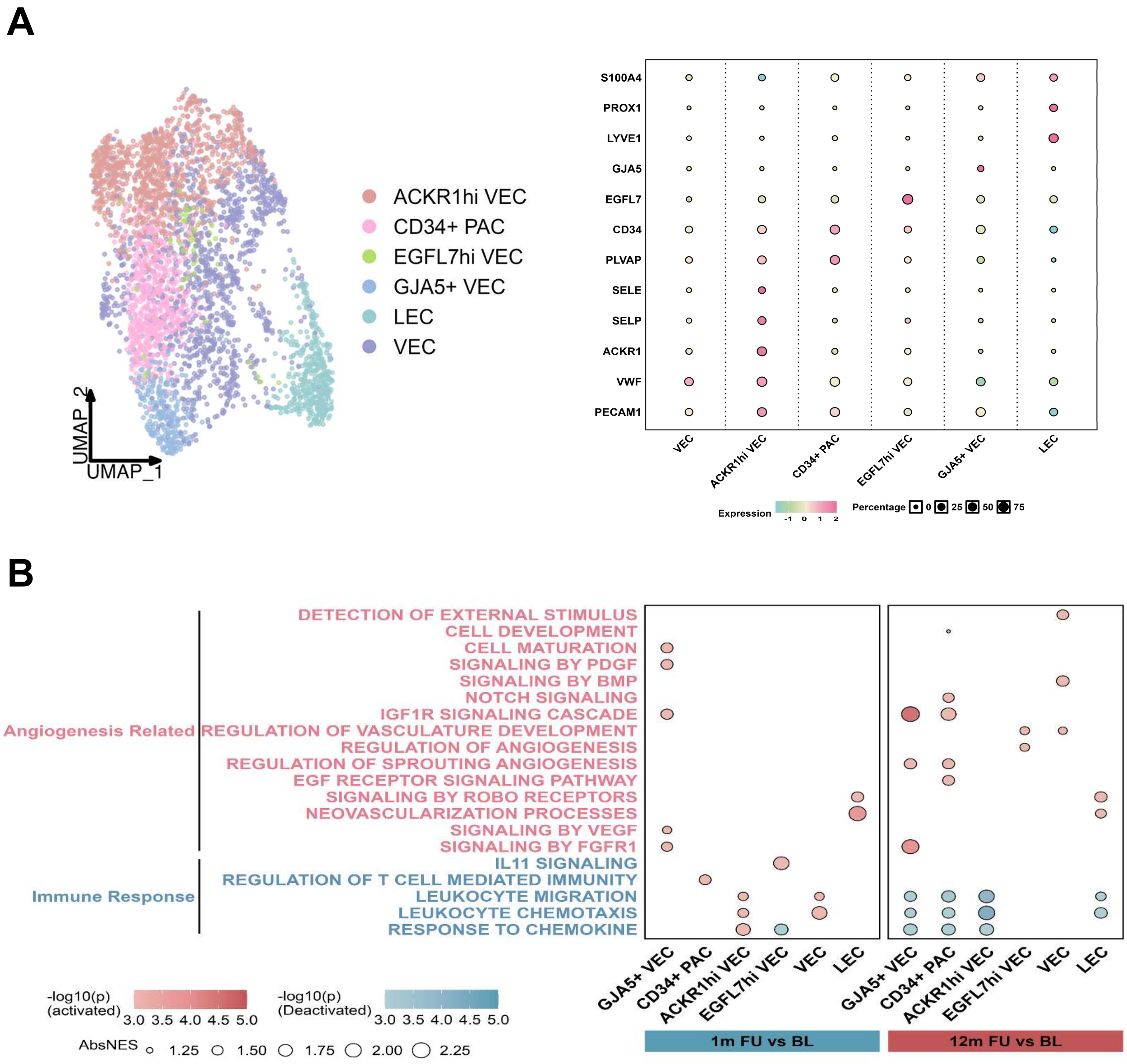

Supplementary Figure 19: Endothelial cell populations identified by cISH. A UMAP plot shows clustering of endothelial cell detected by cISH, colored by subtype identity. Dot plot characterizes six endothelial cell subpopulations based on the expression of known marker genes, where dot size represents the proportion of expressing cells and dot color indicates the average expression level. **B** Dot plot showing enriched pathways detected by FGSEA across different endothelial cell subtypes. Pathways are grouped into two functional categories—Angiogenesis-related, and immune response—and are colored accordingly. Dot size represents the absolute normalized enrichment score (AbsNES), while dot color reflects the log-transformed p-value. Red dots indicate activated pathways, and blue dots indicate deactivated pathways.

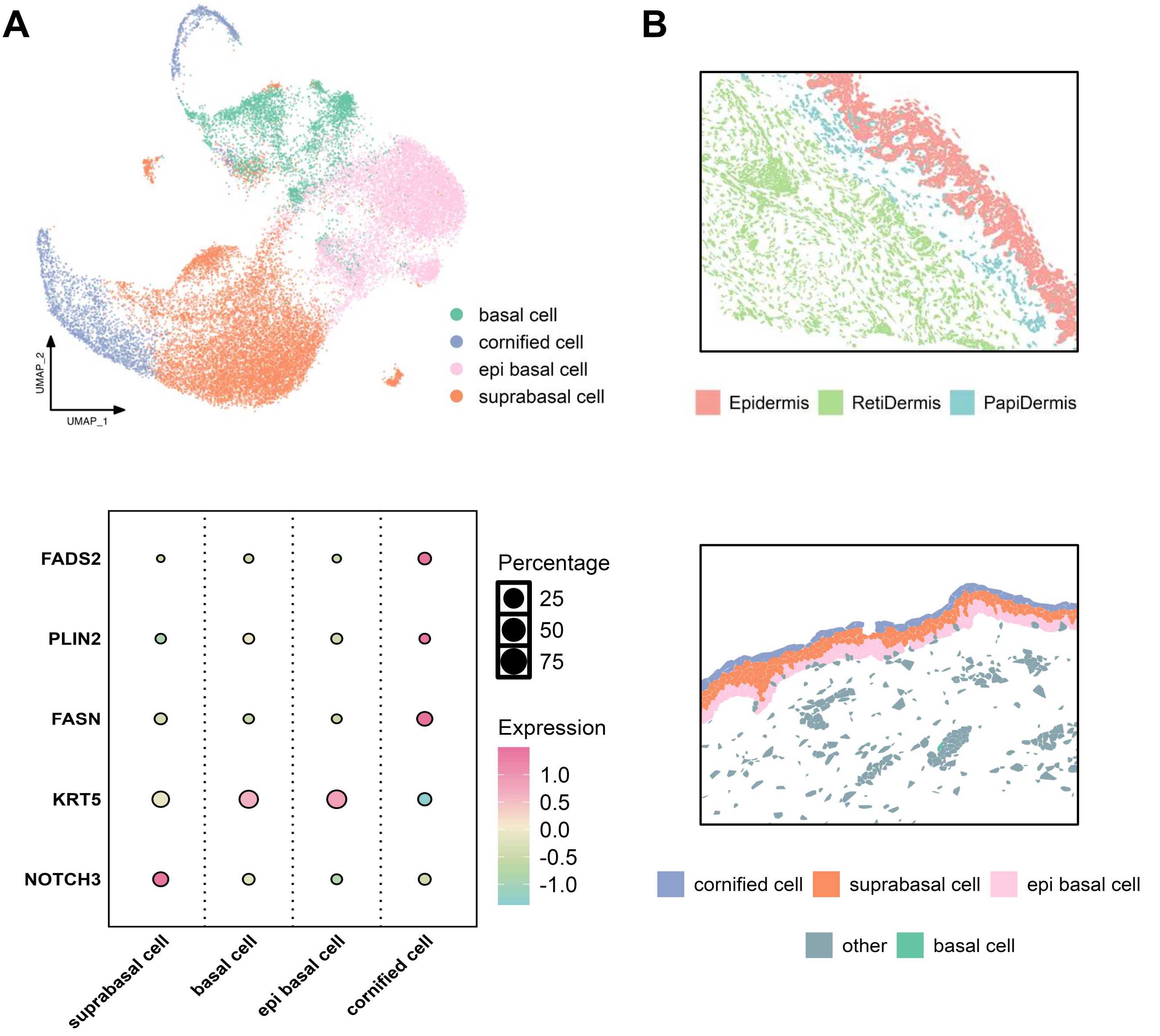

Supplementary Figure 20: Epithelial populations identified by cISH. A UMAP plot shows clustering of epithelial cells detected by cISH, including BL and all follow-up samples (n=23 samples). The color represents the identity of the epithelial population. Dot plot characterizes four keratinocyte subpopulations based on the expression of known marker genes, where dot size represents the proportion of expressing cells and dot color indicates the average expression level. **B** Spatial plot (patient 2) showing the distribution of cells across three regions: epidermis, papillary dermis (PapiDerm), defined as cells residing within 100 μm from the epidermal layer, and reticular dermis (RetiDerm), representing the remaining cells beyond this threshold. Spatial plot (patient 5) depicting the distribution of keratinocyte subpopulations, including segregated epidermal basal (epibasal) cells, based on their spatial location within the tissue.

		CD19-CAR T-cell therapy										
		Patient 1	Patient 2	Patient 3	Patient 4	Patient 5	Patient 6	Patient 7	Patient 8	Patient 9	Patient 10	Patient 11
Demogr	Age (years)	60	36	37	47	55	29	23	34	20	54	62
	Sex	male	male	female	male	male	female	female	male	female	male	male
	Disease duration (months)	23	30	15	134	42	47	9	5	53	71	73
AB profile	ANA	1:320	1:10000	1:10000	1:1000	1:3200	1:3200	1:10000	1:10000	1:3200	1:3200	1:3200
	Anti-Scl70	-	+++	+	+++	+++	+++	+++	+++	+++	-	+++
	Anti-RNAP III	+++	-	-	-	-	-	-	-	-	-	-
	Other	-	-	-	-	ro52	-	-	pm-scl75	-	pm-scl75	-
Skin	diffuse cutaneous	+	+	+	+	+	+	+	+	+	+	+
	mRSS (0-51)	24	27	32	17	35	25	19	18	16	23	27
	Tendon friction rubs	+	+	-	-	-	-	-	-	-	-	-
	Digital ulcerations	-	+	+	-	+	-	+	-	+	-	+
Lung involvement		+	+	+	+	+	+	+	+	+	+	+
Heart involvement		+	+	-	-	+	-	+	-	-	-	+
EUSTAR AI		4.8	10.0	5.3	3.9	4.8	2.5	7.8	5.5	5.3	6.3	4.8
Previous treatment		MMF, MTX	MMF, Gluc, HCQ	MMF, MTX, Toc, RTX	MMF, Cyc, Nint	MMF, MTX, Gluc, RTX,	MMF, MTX, RTX	MTX, MMF, Nint	MMF, Nint	MTX, Gluc, MMF, Toc, RTX	Gluc, MTX, RTX	MTX, Toc, Aba, Nint, MMF, RTX, IVIG
Previous/current vasodilators		Sil	Nif, Ilo	Sil, Alpro, Bos		Bos	Sil, Bos	Alpro, Tada	Tada, Bos			Ilo, Bos

		Standard-of-care treatments									Natural disease course
		Patient 1	Patient 2	Patient 3	Patient 4	Patient 5	Patient 6	Patient 7	Patient 8		Patient 1
Demogr	Age (years)	44	62	49	45	67	34	23	49		65
	Sex	female	female	male	male	male	male	female	male		female
	Disease duration (months)	2	120	12	53	21	5	9	156		5
AB profile	ANA	1:10000	1:1000	1:1000	1:10000	1:10000	1:1000	1:10000	1:1000		1:3200
	Anti-Scl70	-	+++	+++	+++	-	+++	+++	-		
	Anti-RNAP-III	-	-	-	-	-	-	-	-		+++
	Other	pm - scl75/100	-	-	-	pm-scl75/100	-	-	pm-scl75/100		-
Skin	diffuse cutaneous	-	+	+	+	+			+		-
	mRSS (0-51)	9	14	16	10	16	18	19	23		12
	Tendon friction rubs	-	-	-	-	-	-	-	-		-
	Digital ulcerations	-	-	-	-	-	-	+	-		-
Lung involvement		+	+	+	+	+	+	+	+		+
Heart involvement		-	-	-	-	+	-	+	-		-
EUSTAR AI		2.3	4.8	6.1	3.3	2.5	3.7	6.8	4.0		2.0
Previous/current* treatment		MMF*	MMF*	Gluc*, MTX*	Gluc*, MTX, Aza, RTX, Nint*	Nint, MMF*	MMF*, Nint*	MTX, MMF*, Nint*	MTX, Gluc, MMF*		Nint
Previous/current vasodilators		Nif	Bos, Sil, Nif, Alpro	Nif			Tada, Bos	Alpro, Tada	Nif, Alpro, Fluo, Sil		

Supplementary table 1: Baseline characteristics of patients with diffuse systemic sclerosis before CD19-targeting CAR T cell therapy, standard-of-care (SOC) treatments or with natural disease course (NDC). As a standard-of-care control group (SOC), we examined serial skin samples from patients who met the inclusion criteria and had received standard-of-care treatments such as mycophenolate mofetil, tocilizumab, rituximab, or methotrexate. In addition, we included samples from a patient with dcSSc who had achieved disease stabilization under standard-of-care therapy and were no longer receiving immunosuppression; these were designated as the "natural disease course group" (NDG). AB: antibody, ANA: antinuclear antibodies; Anti-Scl70: anti-topoisomerase I antibody; Anti-RNAP-III: anti-ribonucleid acid polymerase III; Demogr: demographic data. IgG: Immunoglobulin G; mRSS: modified Rodnan Skin Score; FVC: Forced vital capacity; DLCO: diffusing capacity of the lung for carbon monoxide; PASP: pulmonary artery systolic pressure; RA: right atrium; NT-proBNP: N-terminal prohormone of brain natriuretic peptide; eGFR: estimated glomerular filtration rate: MMF: Mycophenolate mofetil; MTX: Methotrexate; HCQ: Hydroxychloroquine; RTX: Rituximab; Toc: Tocilizumab; Cyc: Cyclophosphamid; Gluc: Glucocorticoids.; Sil: Sildenafil; Nif: Nifedipin; Ilo: Iloprost; Alpro: Alprostadil; Bos: Bosentan; Tada: Tadalafil, Aba: Abatacept; Nint: Nintedanib; IVIG: intravenous immunoglobulins; Aza: Azathioprin; Fluo: Fluoxetin; *=ongoing treatment at timepoint of baseline.
