# Supplementary material for: Deep phenotyping of skin tissue remodeling in patients with systemic sclerosis treated with CD19-CAR T cells
Source: Nat Commun. 2026 May 23;17:4640. doi: 10.1038/s41467-026-72817-7 (PMC13201536; doi:10.1038/s41467-026-72817-7)
Supplement: Supplementary file 3 — Reporting Summary [file 41467_2026_72817_MOESM3_ESM.docx]

Ruth O Allen1


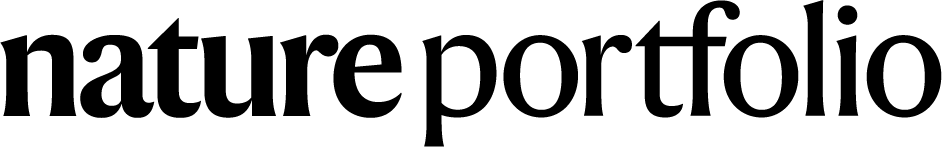
Corresponding author(s): Christina Bergmann

Last updated by author(s): March, 10, 2026

Reporting Summary

Nature Portfolio wishes to improve the reproducibility of the work that we publish. This form provides structure for consistency and transparency in reporting. For further information on Nature Portfolio policies, see our Editorial Policies and the Editorial Policy Checklist.

Please do not complete any field with "not applicable" or n/a. Refer to the help text for what text to use if an item is not relevant to your study. For final submission: please carefully check your responses for accuracy; you will not be able to make changes later.

## Statistics

For all statistical analyses, confirm that the following items are present in the figure legend, table legend, main text, or Methods section.

n/a


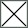

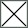

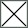


Confirmed


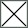
 The exact sample size (*n*) for each experimental group/condition, given as a discrete number and unit of measurement


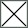
 A statement on whether measurements were taken from distinct samples or whether the same sample was measured repeatedly The statistical test(s) used AND whether they are one- or two-sided

*Only common tests should be described solely by name; describe more complex techniques in the Methods section.*


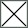
 A description of all covariates tested


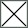
 A description of any assumptions or corrections, such as tests of normality and adjustment for multiple comparisons

A full description of the statistical parameters including central tendency (e.g. means) or other basic estimates (e.g. regression coefficient) AND variation (e.g. standard deviation) or associated estimates of uncertainty (e.g. confidence intervals)

For null hypothesis testing, the test statistic (e.g. *F*, *t*, *r*) with confidence intervals, effect sizes, degrees of freedom and *P* value noted

1

nature portfolio | reporting summary

*April 2023*

*Give P values as exact values whenever suitable.*


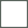
 For Bayesian analysis, information on the choice of priors and Markov chain Monte Carlo settings


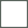
 For hierarchical and complex designs, identification of the appropriate level for tests and full reporting of outcomes
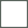
 Estimates of effect sizes (e.g. Cohen's *d*, Pearson's *r*), indicating how they were calculated

*Our web collection on statistics for biologists contains articles on many of the points above.*

## Software and code

Policy information about availability of computer code Data collection

Multi-beam OCT scanner VivoSight Dx (Michelson Diagnostics Ltd., Maidstone, Kent, UK); CARE Dose4D algorithm (Siemens, Erlangen, Germany); Nanozoomer S60v2MD slide scanner (Hamamatsu Photonics, Herrsching am Ammersee; NDPView2 software (Hamamatsu Photonics); Curvealign V4.0 Beta (MATLAB); Nikon Eclipse 80i microscope (Nikon, Tokyo, Japan); NovaSeq-6000 platform (Illumina); Cytometry by time-of-flight (CyTOF) (Helios, Standard Biotools, South San Francisco, Ca, USA); Hyperion Imaging System (Standard Biotools, South San Francisco, Ca, USA); Xenium Onboard Analysis (10X Genomics, version 3.0). Clinical data collection was conducted using Microsoft Excel.

Data analysis

The IMC data preprocessing and cell segmentation was done using the Steinbock (v16.8) pipeline [(https://bodenmillergroup.github.io/steinbock/).](https://bodenmillergroup.github.io/steinbock/)) All downstream analysis were done using the R package imcRtools (v1.12.0) [(https://github.com/BodenmillerGroup/imcRtools).](https://github.com/BodenmillerGroup/imcRtools)) The cISH data analysis was performed using Seurat [(https://github.com/satijalab/seurat),](https://github.com/satijalab/seurat)) sccomp [(https://github.com/MangiolaLaboratory/sccomp),](https://github.com/MangiolaLaboratory/sccomp)) fgsea (https:// github.com/alserglab/fgsea), decoupleR [/https://github.com/saezlab/decoupleR),](https://github.com/saezlab/decoupleR)) spatialEco [(https://github.com/jeffreyevans/spatialEco),](https://github.com/jeffreyevans/spatialEco)) scvi-tools [(https://github.com/scverse/scvi-tools),](https://github.com/scverse/scvi-tools)) and CellChat [(https://github.com/jinworks/CellChat)](https://github.com/jinworks/CellChat)) R packages.

For manuscripts utilizing custom algorithms or software that are central to the research but not yet described in published literature, software must be made available to editors and reviewers. We strongly encourage code deposition in a community repository (e.g. GitHub). See the Nature Portfolio guidelines for submitting code & software for further information.

2

nature portfolio | reporting summary

*April 2023*

## Data

Policy information about availability of data

All manuscripts must include a data availability statement. This statement should provide the following information, where applicable:

- Accession codes, unique identifiers, or web links for publicly available datasets
- A description of any restrictions on data availability
- For clinical datasets or third party data, please ensure that the statement adheres to our policy

All the data supporting the findings of this study are available within the article or the supplementary material provided. All source data can be found in the accompanying Source data file. Bulk RNAseq, cISH and IMC data are available upon reasonable request.

## Research involving human participants, their data, or biological material

Policy information about studies with human participants or human data. See also policy information about sex, gender (identity/presentation), and sexual orientation and race, ethnicity and racism.

Reporting on sex and gender

The sex of the patients involved in the study was registered as indicated in the electronic medical record system based on health insurance information. A total of 11 patients were involved in the study, 4 (36,36%) of them were female and 7 (63,63%) were male. No significant differences between sexes were observed in the parameters that were analyzed in the study.

Reporting on race, ethnicity, or other socially relevant groupings

All patients enrolled in the study were caucasian.

Population characteristics

Eleven dcSSc patients were enrolled in the study, with disease durations ranging from 5 to 134 months and ages ranging from 23 to 62 years old. All eleven patients received CD19-CAR T-cell therapy.

Recruitment

Patients were recruiting based on the following criteria: positivity for anti-Topoisomerase I (anti-Scl70) or anti-RNA polymerase III antibodies, and active systemic sclerosis as defined by either (1) disease duration of less than 7 years (since first non-Raynaud symptom); (2) modified Rodnan Skin Score (mRSS) 10–35 at screening; (3) elevated acute phase reactants (C-reactive protein ≥ 6 mg/L, erythrocyte sedimentation rate ≥28 mm/1 h, or platelet count ≥330 109/L); or (4) mRSS increase by 3 points or more or involvement of a new body area, or mRSS increase by 2 points or more in one body area, or 1 or more tendon friction rub instances over 6 months, or signs of progressive lung disease as defined by the criteria used in the INBUILD study, or new onset of interstitial lung disease within 6 months before baseline (≥10% disease extent, based on CT scan). Eligible patients had insufficient response or intolerance to at least two standard-of-care treatments, including mycophenolate mofetil, azathioprine, cyclophosphamide, nintedanib, methotrexate, rituximab, or tocilizumab.

Ethics oversight

All patients enrolled in the study gave informed consent for all the procedures and the data sharing according to CARE guidelines and in compliance with the principles of the Declaration of Helsinki. The collection and use of patient data and biomaterial are covered by license 334_18 B of the Institutional Review Board of the University Hospital Erlangen. All procedures were performed in accordance with the Good Clinical Practice guidelines of the International Council for Harmonization. The CASTLE study was approved by the ethics committee of Friedrich-Alexander University Erlangen (22-168-Az). The primary and secondary outcomes of this ongoing study will be published on the completion of the trial.

Note that full information on the approval of the study protocol must also be provided in the manuscript.

# Field-specific reporting

Please select the one below that is the best fit for your research. If you are not sure, read the appropriate sections before making your selection.


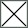
 Life sciences
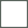
 Behavioural & social sciences
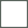
 Ecological, evolutionary & environmental sciences

For a reference copy of the document with all sections, see nature.com/documents/nr-reporting-summary-flat.pdf

# Life sciences study design

All studies must disclose on these points even when the disclosure is negative. Sample size

The study's primary objectives being descriptive and mechanistic, a statistically-powered sample size calculation was not required and therefore not performed. This study was an exploratory trial designed to characterize biological changes and generate hypotheses, rather than test predefined effect sizes. Eleven participants were included based on feasibility and the number of eligible patients meeting the inclusion criteria during the recruitement period. This sample size was sufficient to detect qualitative and interindividual trends accross time points and the available number of previously collected control samples was sufficient to support the comparative analyses conducted.

Data exclusions

No data were excluded from the analyses.

3

nature portfolio | reporting summary

*April 2023*

Replication

The data reported in this manuscript were reproduced with at least 3 biological replicates for each sample in in vitro and microscopy procedures.

Randomization

Any subgrouping used in the study was based on predefined clinical assessment and not on random assignment. Participants were therefore not randomized. All enrolled patients met predefined inclusion criteria related to diagnosis, clinical symptoms, disease-specific parameters and response to previous treatments. Considering that this study was an exploratory trial, participants were assigned to a single observational cohort. No covariate-based allocation or stratification was required. Relevant covariates (e.g. age, disease duration and severity at baseline) were recorded and considered in downstream analyses when appropriate, and formal covariate controls at the allocation step were not applicable. Control samples were obtained from previously collected, independent specimens that were not part of the trial and were used to provide baseline comparison data.

Blinding

Considering the exploratory nature of the study, laboratory-based investigators conducting data collection and analysis were blinded to group assignments, but not the clinical staff or study participants.

# Reporting for specific materials, systems and methods

We require information from authors about some types of materials, experimental systems and methods used in many studies. Here, indicate whether each material, system or method listed is relevant to your study. If you are not sure if a list item applies to your research, read the appropriate section before selecting a response.

Materials & experimental systems Methods


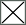

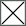

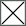

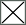

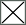


n/a Involved in the study Antibodies Eukaryotic cell lines

Palaeontology and archaeology Animals and other organisms Clinical data

Dual use research of concern

Plants


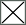

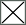

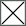


n/a Involved in the study

ChIP-seq

Flow cytometry

MRI-based neuroimaging

## Antibodies

Antibodies used

All antibodies used in this study are listed in the provided supplementary materials.

Validation

All used antibodies were first validated by immunofluorescence staining on human skin sections using serial dilutions, alongside a no-primary antibody as negative control. For Imaging Mass Cytometry (IMC), the same validation procedure was performed prior to metal conjugation. Only the antibodies for which expression patterns were consistent with those described in literature passed the quality criteria. A titration for all antibodies was done for optimal dilution. Antibodies were used as instructed by the suppliers.

## Clinical data

Policy information about clinical studies

All manuscripts should comply with the ICMJE guidelines for publication of clinical research and a completed CONSORT checklist must be included with all submissions.

Clinical trial registration

CASTLE phase 1/2 study: NCT06347718; EudraCT-Nr. 2022-001366-35

Study protocol

The full protocol can be accessed in the Methods section of the manuscript supplementary material.

Data collection

As indicated in the methods section of the supplementary material in the manuscript, MRSS was documented at baseline, one month, six months and twelve months after CAR19-CAR T-cell treatment. Raynaud’s phenomenon was assessed using a patient-reported diary on the number of attacks, duration and pain intensity of Raynaud symptoms at three consecutive days at baseline as well as three months after CD19-CAR T-cell therapy. Lung function parameters including forced vital capacity (FVC) and diffusing capacity for carbon monoxide (DLCO) were assessed at baseline and at least every six months throughout the follow up period.

Capillaroscopy was performed at baseline and one, three, six months after therapy, assessing the number of capillaries per millimeter in multiple pictures taken from 2-4 fingers at each time point. Optical coherence tomography (OCT) was performed on the back of the hand before and two months after the application of CD19-CAR T-cell therapy using the multi-beam OCT scanner VivoSight Dx (Michelson Diagnostics Ltd., Maidstone, Kent, UK). A scan of an area of 6 mm x 6 mm provides 120 cross-sectional images (6 mm x 2 mm) with a lateral image resolution of < 7.5 µm, an axial resolution of < 10 µm and a depth of 1.5 mm. Blood vessels were visualized using the dynamic mode, which exploits repeated scans of the same area to detect changes, which are then automatically color coded and correspond to blood flow in dermal vessels.

Outcomes

Primary and secondary outcome measures were described in the methods section of the manuscript.

4

nature portfolio | reporting summary

*April 2023*

## Plants

Seed stocks

n/a

Novel plant genotypes

n/a

Authentication

n/a


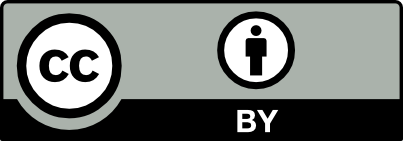
This checklist template is licensed under a Creative Commons Attribution 4.0 International License, which permits use, sharing, adaptation, distribution and reproduction in any medium or format, as long as you give appropriate credit to the original author(s) and the source, provide a link to the Creative Commons license, and indicate if changes were made. The images or other third party material in this article are included in the article's Creative Commons license, unless indicated otherwise in a credit line to the material. If material is not included in the article's Creative Commons license and your intended use is not permitted by statutory regulation or exceeds the permitted use, you will need to obtain permission directly from the copyright holder. To view a copy of this license, visit <http://creativecommons.org/licenses/by/4.0/>
