# Supplementary material for: Deep phenotyping of skin tissue remodeling in patients with systemic sclerosis treated with CD19-CAR T cells
Source: Nat Commun. 2026 May 23;17:4640. doi: 10.1038/s41467-026-72817-7 (PMC13201536; doi:10.1038/s41467-026-72817-7)
Supplement: Supplementary file 10 — Description of Additional Supplementary Files [file 41467_2026_72817_MOESM10_ESM.pdf]

Description of Additional Supplementary Files

Supplementary Data 1

Description: Results of the CAR T response score across cell types.

Supplementary Data 2

Description: Clinical treatment indications in the SoC group.

Supplementary Data 3

Description: Supplementary Clinical Information and Sample overview of the CAR T group.

Supplementary Data 4

Description: Genes and fold changes used for computing the CAR T response score.

Supplementary Data 5

Description: Genomic Sequences targeted by the probes for cISH analysis.
